# Improving bowel preparation for colonoscopy with a smartphone application driven by artificial intelligence

**DOI:** 10.1038/s41746-023-00786-y

**Published:** 2023-03-14

**Authors:** Yan Zhu, Dan-Feng Zhang, Hui-Li Wu, Pei-Yao Fu, Li Feng, Kun Zhuang, Zi-Han Geng, Kun-Kun Li, Xiao-Hong Zhang, Bo-Qun Zhu, Wen-Zheng Qin, Sheng-Li Lin, Zhen Zhang, Tian-Yin Chen, Yuan Huang, Xiao-Yue Xu, Jing-Zheng Liu, Shuo Wang, Wei Zhang, Quan-Lin Li, Ping-Hong Zhou

**Affiliations:** 1 Endoscopy Center and Endoscopy Research Institute, Zhongshan Hospital, Fudan University, Shanghai, China; 2 Shanghai Collaborative Innovation Center of Endoscopy, Shanghai, China; 3 Department of Gastroenterology, Zhengzhou Central Hospital, Henan, China; 4 Endoscopy Center, Central Hospital of Minhang District, Shanghai, China; 5 Department of Gastroenterology, Xian Central Hospital, Shaanxi, China; 6 Digital Medical Research Center, School of Basic Medical Sciences, Fudan University, Shanghai, China; 7 Shanghai Key Laboratory of Medical Imaging Computing and Computer Assisted Intervention, Shanghai, China; 8 Department of Biostatistics, School of Public Health, Fudan University, Shanghai, China

**Keywords:** Patient education, Colonoscopy

## Abstract

Optimal bowel preparation is a prerequisite for a successful colonoscopy; however, the rate of inadequate bowel preparation remains relatively high. In this study, we establish a smartphone app that assesses patient bowel preparation using an artificial intelligence (AI)-based prediction system trained on labeled photographs of feces in the toilet and evaluate its impact on bowel preparation quality in colonoscopy outpatients. We conduct a prospective, single-masked, multicenter randomized clinical trial, enrolling outpatients who own a smartphone and are scheduled for a colonoscopy. We screen 578 eligible patients and randomize 524 in a 1:1 ratio to the control or AI-driven app group for bowel preparation. The study endpoints are the percentage of patients with adequate bowel preparation and the total BBPS score, compliance with dietary restrictions and purgative instructions, polyp detection rate, and adenoma detection rate (secondary). The prediction system has an accuracy of 95.15%, a specificity of 97.25%, and an area under the curve of 0.98 in the test dataset. In the full analysis set (*n* = 500), adequate preparation is significantly higher in the AI-driven app group (88.54 vs. 65.59%; *P* < 0.001). The mean BBPS score is 6.74 ± 1.25 in the AI-driven app group and 5.97 ± 1.81 in the control group (*P* < 0.001). The rates of compliance with dietary restrictions (93.68 vs. 83.81%, *P* = 0.001) and purgative instructions (96.05 vs. 84.62%, *P* < 0.001) are significantly higher in the AI-driven app group, as is the rate of additional purgative intake (26.88 vs. 17.41%, *P* = 0.011). Thus, our AI-driven smartphone app significantly improves the quality of bowel preparation and patient compliance.

## Introduction

Recent global estimates of cancer incidence and mortality place colorectal cancer (CRC) as the fourth most prevalent and second deadliest cancer worldwide^1^. The combination of a well-defined precursor lesion and a long preclinical course makes CRC an ideal candidate for cancer prevention screening^2^, the principal method of which is colonoscopy. However, many factors can impact the accuracy of colonoscopy, especially the quality of bowel preparation^3^. Optimal bowel preparation, a prerequisite for a successful colonoscopy, includes an appropriate volume of purgatives, appropriate timing of purgative consumption, and at least 3 days of dietary restrictions^4,5^. Consequently, the rate of inadequate bowel preparation is as high as 20–25%^6,7^. Inadequate bowel preparation often results from an unwillingness to follow the preparation instructions, difficulties following the prescribed diet, or inability to tolerate the full course of purgatives^8^. Therefore, it is necessary to reinforce patient education on bowel preparation.

Enhanced education significantly improves the quality of bowel preparation for colonoscopy and increases patient willingness to undergo bowel preparation^9^. Bowel preparation education can be reinforced by visual aids and directed reminders. Visual aids include patient educational booklets^10^, cartoon visual aids^11^, and nurse-delivered education with brochures^12^. Directed reminders include short messages^13^, telephone-based instructions^14^, social media platforms^15^, and smartphone apps^16,17^.

The existing education approaches are effective but have certain drawbacks. The most important limitation is that they often have fixed schedules and educational content that does not account for differences in bowel preparation status (i.e., adequate or inadequate). Ideally, patients should receive personalized reminder messages or educational content reflecting their current bowel preparation status. Furthermore, patients often have difficulty evaluating the adequacy of their bowel preparation. Visual aids, such as photographs of “clean” and “dirty” feces in the toilet, may be useful, but they cannot cover all situations and are often difficult for older patients to use. Accurate, real-time evaluation of bowel preparation status would be useful in generating personalized instructions.

Artificial intelligence (AI) has the potential to overcome certain clinical/human obstacles by enabling real-time diagnosis or guidance in many fields of medicine, including endoscopy^18–22^, where it may be the solution for real-time evaluation of outpatient bowel preparation. Our preliminary experiments reveal that AI technology can be used to evaluate bowel preparation status according to the appearance of feces in the toilet, and an AI-driven smartphone app may provide more personalized and accurate enhanced instructions to improve bowel preparation compared with traditional education approaches. The AI system acts as an evaluator, the results of which inform the smartphone app’s personalized reminders.

In this study, we create an AI-based bowel preparation prediction system for outpatients scheduled for colonoscopy to predict bowel preparation quality in real time by evaluating photos of feces in the toilet. Based on this prediction system, we create an AI-driven smartphone app to provide personalized enhanced instructions to improve the patients’ bowel preparation. After creating the app, we conduct a prospective multicenter study to evaluate the impact of this AI-driven smartphone app on the quality of bowel preparation in colonoscopy outpatients.

## Results

### Performance of the AI-based bowel preparation evaluation system

After 350 epochs of training, the model converged well and showed satisfactory performance on the test dataset (Supplementary Information 1). Although ShuffleNet v2 is a lightweight network, it achieved an accuracy of 95.15% (Supplementary Table 2). The specificity was 97.25%, indicating that the model can accurately identify photographs that represent inadequate bowel preparation. The area under the receiver operating characteristics curve (AUC) was 0.98 in the test dataset (Supplementary Fig. 5).

### Patient characteristics

**Fig. 1:**
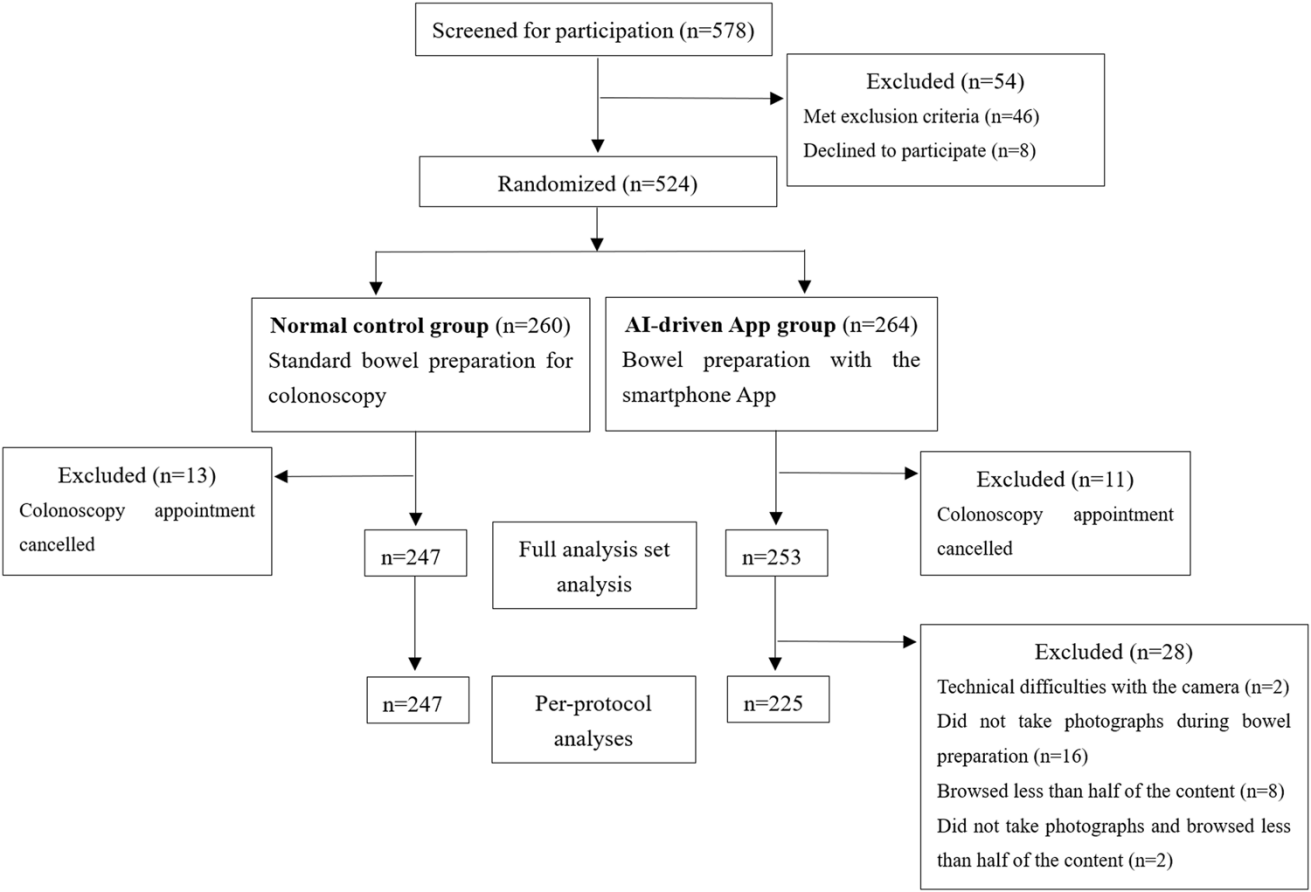
Flowchart of the clinical trial. AI artificial intelligence, App application.

Overall, 578 patients were scheduled for colonoscopy examination during the study period (Table 1). After excluding 54 patients who met the exclusion criteria or declined to participate, 524 eligible individuals were randomized to the control group or the AI-driven app group. Twenty-four individuals cancelled their colonoscopy appointment and did not reschedule. Ultimately, 500 participants—247 in the control group and 253 in the AI-driven app group—were enrolled and included in the full analysis set (FAS) (Fig. 1). After excluding patients who did not use the app correctly, 225 patients were included in the AI-driven app group (per-protocol set [PPS]).

**Table 1:** Baseline characteristics of the total analysis population.

Characteristic	AI-driven app group (*n* = 253)	Control group (*n* = 247)
Age, years, mean ± SD	51.40 ± 12.56	53.35 ± 14.03
Men, *n* (%)	126 (49.80)	114 (46.15)
Body mass index, kg/m^2^, mean ± SD	23.60 ± 3.86	23.68 ± 3.45
ASA class, *n* (%)		
I	186 (73.52)	172 (69.64)
II	67 (26.48)	75 (30.36)
Indication, *n* (%)		
CRC screening	72 (28.46)	72 (29.15)
Surveillance after the previous colonoscopy	30 (11.86)	31 (12.55)
Diagnostic	151 (59.68)	144 (58.30)
Prior colonoscopy, *n* (%)	92 (36.36)	94 (38.06)
Previous surgery (abdominal or pelvic), *n* (%)	45 (17.79)	54 (21.86)
Medical history, *n* (%)		
Diabetes mellitus	13 (5.14)	15 (6.07)
Hypertension	30 (11.86)	40 (16.24)
Chronic constipation	50 (19.76)	51 (20.65)
Liver cirrhosis	7 (2.77)	12 (4.86)
Education level, *n* (%)		
University graduation	137 (54.15)	123 (49.80)
High school graduation	71 (28.06)	70 (28.34)
Middle or elementary school	45 (17.79)	54 (21.86)
Marital status, *n* (%)		
Single/widowed	28 (11.07)	22 (8.91)
Married/partnership	225 (88.93)	225 (91.09)

*ASA* American Society of Anesthesiologists, *CRC* colorectal cancer, *SD* standard deviation.

### Outcomes of bowel preparation and colonoscopy

In the FAS analyses (Table 2), the rate of adequate bowel preparation (Boston Bowel Preparation Scale [BBPS] score≥6) was significantly higher in the AI-driven app group than in the control group (88.54 vs. 65.59%, *P* < 0.001). The PPS analyses revealed similar results for the primary outcome: 89.78% of patients in the AI-driven app group and 65.59% of patients in the control group achieved adequate bowel preparation (*P* < 0.001). Both the FAS and PPS analyses showed that the rate of excellent bowel preparation (BBPS score≥8) was significantly higher in the AI-driven app group than in the control group (FAS: 27.67 vs. 19.84%, *P* = 0.040; PPS: 28.44 vs. 19.84%, *P* = 0.029).

**Table 2:** The rate of adequate and excellent bowel preparation.

Outcomes	AI-driven app group		Control group		Rate difference	*P* value
	*N*	*n*(%, 95%CI)	*N*	*n*(%, 95%CI)	%; 95%CI	
**Rate of adequate bowel preparation (primary outcome)**						
FAS	253	224 (88.54, 83.95–92.19)	247	162 (65.59, 59.30–71.49)	22.95;15.43–30.20	<0.001
PPS	225	202 (89.78, 85.06–93.41)	247	162 (65.59, 59.30–71.49)	24.19;16.57–31.42	<0.001
**Rate of excellent bowel preparation**						
FAS	253	70 (27.67, 22.25–33.62)	247	49 (19.84, 15.05–25.36)	7.83;0.07–15.45	0.040
PPS	225	64 (28.44, 22.65–34.82)	247	49 (19.84, 15.05–25.36)	8.61;0.59–16.57	0.029

*FAS* full analysis set, *PPS* per-protocol set, *CI* confidence interval.

The mean BBPS score was 6.74 ± 1.25 in the AI-driven app group and 5.97 ± 1.81 in the control group (*P* < 0.001) (Table 3). The BBPS scores were also significantly higher in the AI-driven app group for each colon segment (left, transverse, and right). The cecal intubation rate was 100% in both groups. The AI-driven app group had a shorter cecal intubation time (5.06 ± 2.07 min vs. 5.86 ± 2.85 min, *P* < 0.001). The mean withdrawal time and colonoscopy time were similar between the groups. No serious complications or adverse events were reported during the study period. Overall, 381 polyps were detected within the study, including 223 adenomas and 42 advanced adenomas. The polyp detection rate (PDR), adenoma detection rate (ADR), and advanced adenoma detection rate (aADR) were not significantly different between the groups.

**Table 3:** Effect of AI-driven app on the outcome of bowel preparation and colonoscopy.

Outcomes	AI-driven app group (*n* = 253)	Control group (*n* = 247)	Difference (95% CI)	*P* value
Total BBPS score, mean ± SD	6.74 ± 1.25	5.97 ± 1.81	0.77 (0.49–1.04)	<0.001
BBPS score in different colon segments, mean ± SD				
Right	2.07 ± 0.65	1.70 ± 0.84	0.37 (0.24–0.50)	<0.001
Transverse	2.36 ± 0.57	2.15 ± 0.72	0.21 (0.09–0.32)	<0.001
Left	2.30 ± 0.56	2.12 ± 0.66	0.18 (0.07–0.29)	<0.001
Polyp detection rate	114 (45.06%)	98 (39.68%)	5.38% (−3.54%–14.19%)	0.223
Adenomas detected rate	70 (27.67%)	56 (22.67%)	5.00% (−2.90%–12.80%)	0.198
Advanced adenomas detected rate	16 (6.32%)	10 (4.05%)	2.27% (−2.00%–6.55%)	0.252
Successful cecal intubation	253 (100%)	247 (100%)	-	-
Cecal intubation time (min), mean ± SD	5.06 ± 2.07	5.86 ± 2.85	−0.80 (−1.23– −0.36)	<0.001
Withdrawal time (min), mean ± SD	7.63 ± 3.68	7.36 ± 3.30	0.27 (−0.35–0.88)	0.392
Colonoscopy time (min), mean ± SD	12.70 ± 4.32	13.22 ± 4.60	−0.52 (−1.31–0.26)	0.188

*CI* confidence interval, *SD* standard deviation, *BBPS* Boston bowel preparation scale.

### Bowel preparation process

The effects of app usage on the bowel preparation process are shown in Table 4. The FAS analyses revealed no significant differences in scheduled colonoscopy time between the groups (*P* = 0.082). Compared with the control group, the AI-driven app group had significantly higher rates of compliance with dietary restrictions (93.68 vs. 83.81%, *P* = 0.001) and purgative instructions (96.05 vs. 84.62%, *P* < 0.001). The proportion of patients who consumed additional polyethylene glycol (PEG; total >3 L) was higher in the AI-driven app group than in the control group (26.88 vs. 17.41%, *P* = 0.011). In the subgroup analysis of patients who consumed additional PEG, bowel preparation was adequate and perfect in 95.59 and 27.94% of patients in the AI-driven app group and only in 65.11 and 13.95% of patients in the control group, respectively.

**Table 4:** Effect of AI-driven app on the procedure of bowel preparation.

Outcomes	AI-driven app group (*n* = 253)	Control group (*n* = 247)	*P* value
Scheduled colonoscopy time, *n* (%)			0.082
8:30–11:30	203 (80.24)	182 (73.68)	
13:30–16:30	50 (19.76)	65 (26.32)	
Compliance with dietary restrictions, *n* (%)	237 (93.68)	207 (83.81)	0.001
Compliance with purgative instructions, *n* (%)	243 (96.05)	209 (84.62)	<0.001
Additional purgative, *n* (%)	68 (26.88)	43 (17.41)	0.011
Willingness to undergo repeat bowel preparation, *n* (%)	5.06 ± 2.07	204 (82.59)	0.002
Sleep quality, *n* (%)			0.006
Worse than usual	153 (60.47)	178 (72.06)	
Same as usual	100 (39.53)	69 (27.94)	

The percentage of patients who reported “good as usual” sleep quality during bowel preparation was higher in the AI-driven app group than in the control group (39.53 vs. 27.94%, *P* = 0.006). The proportion of participants willing to undergo repeat bowel preparation was also higher in the AI-driven app group than in the control group (91.70 vs. 82.59%, *P* = 0.002). In the AI-driven app group, 91.70% of patients reported that they would be willing to use the app again, and 86.16% said they would recommend it to an acquaintance.

In the safety set (SS) analyses, the overall adverse event rates were 28.06 and 22.27% in the app and control groups, respectively (*P* = 0.136). There were no significant differences in abdominal pain, abdominal distention, or nausea/vomiting between the groups (*P* = 0.942).

### Subgroup analysis

**Fig. 2:**
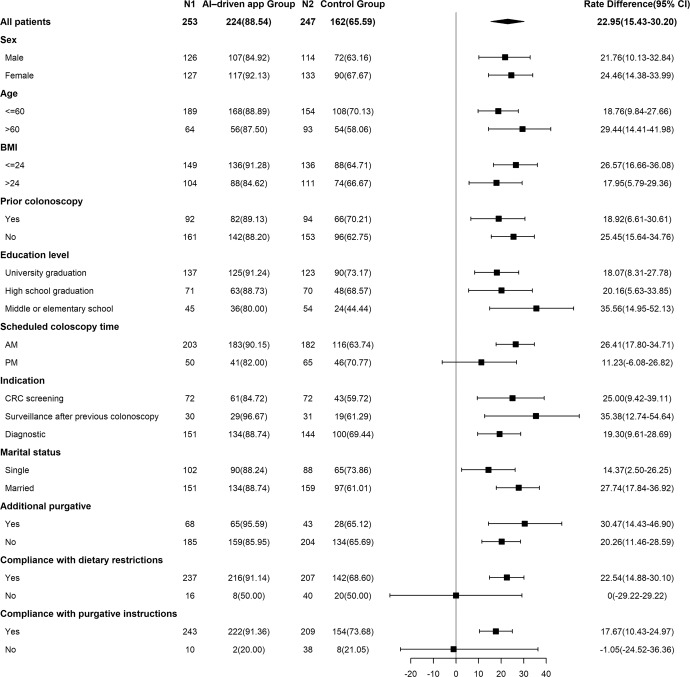
Subgroup analysis of factors associated with the rate of adequate bowel preparation. BMI body mass index, CRC colorectal cancer.

The results of the subgroup analysis (Fig. 2) demonstrate a significantly higher rate of adequate bowel preparation in the AI-driven app group in most subgroups, except among those scheduled for afternoon colonoscopy and those who did not comply with the dietary restrictions or purgative instructions.

## Discussion

In this study, we successfully established an AI-driven smartphone app to aid in colonoscopy bowel preparation. The AI system evaluated bowel preparation status based on photographs of feces in the toilet and provided real-time binary predictions of adequate or inadequate preparation. The app then used these evaluation results to produce personalized messages to improve bowel preparation.

In the clinical trial portion of the study, we found that digitally reinforced patient guidance via the AI-driven app improved the quality of bowel preparation for colonoscopy. Previous studies reported that reinforced education led to satisfactory bowel preparation and achieved a high ADR^14,16^. By contrast, we found no significant difference in PDR and ADR between the groups despite the clear difference in bowel preparation quality. One explanation may be that the sample size (which was based on the rate of adequate bowel preparation) was too small to detect significant differences in these rates.

The AI-driven smartphone app has several advantages over the existing reinforced education methods. Most importantly, the app can deliver personalized patient education with suggestions to improve bowel preparation based on real-time evaluations by the AI system. The recommendation to consume additional purgative may be the most important suggestion associated with adequate bowel preparation. In the AI-driven app group, 26.88% of patients consumed additional purgative based on personalized suggestions provided by the app. By contrast, only 17.41% of patients in the control group took additional purgative based on their own judgment. Previous studies reported that 4-L split-dose (2 + 2 L or 3 + 1 L) PEG was superior to other bowel preparation methods^4,23,24^. However, because of their smaller body size, lower body weight, and different dietary habits, a 4-L PEG volume may be poorly tolerated by the Chinese population^25^. Accordingly, the volume and effectiveness of pre-procedure purgatives must be balanced, considering the exact timing of administration and the selection of patients. Among the patients in the AI-driven app group who took additional purgatives, 95.59 and 27.94% achieved adequate and excellent bowel preparation, respectively. These exceptional results were likely at least partly due to the personalized suggestions delivered by the app.

Another advantage of the app is that it engages patients in the real-time evaluation of their bowel preparation, which is a prerequisite for following suggestions for improvement: when patients know the status of their bowel preparation, they will be more likely to follow suggestions to improve it. The app also improves compliance with dietary restrictions and purgative instructions because it sends patients enhanced education during the bowel preparation process. Both compliance rates were higher in the AI-driven app group in the current study.

A third advantage of our AI system is that patients can use it without an Internet connection, which is important considering the potentially poor network connectivity when individuals are using the toilet. Furthermore, the app stores feces photographs on the user’s smartphone and deletes them after bowel preparation; only the binary prediction results are uploaded to the cloud server for recording. This design avoids transmitting the photographs themselves, thereby guaranteeing patient privacy. To ensure that smartphones could complete the classification task, we utilized the extremely lightweight but efficient convolutional neural network ShuffleNet v2, which runs smoothly on most Android and iOS smartphones and classifies photos within seconds. The group convolution operation and channel shuffle mechanism significantly compressed the size of the neural network and reduced the computing cost^26^.

Compared with the control group, most subgroups performed better with the guidance of the AI-driven smartphone app. The rate difference was enlarged between certain subgroups, however, indicating that some people might benefit more from the AI-driven smartphone-guided bowel preparation method. The rate difference in older adults was higher, so this bowel preparation method might be especially user-friendly for older patients compared with traditional patient education. We noted a similar result among patients with few educational qualifications; the combination of visual aids and directed reminders in the app may be more acceptable than verbal instructions for these individuals. Furthermore, we found the app to be more beneficial for individuals with no prior colonoscopy than for those with colonoscopy experience, which illustrates that this bowel preparation method can be applied to individuals with no bowel preparation experience.

The study has limitations. First, only smartphone users (with Android- or iOS-compatible smartphones) could participate in this study. Therefore, our results may not be applicable to those without regular smartphone access or use. A study published in 2014 using a mobile social media app to improve bowel preparation revealed that among all patients considered for the study, 1039 (44.7%) were excluded because of a lack of convenient access to a smartphone^16^. This may lead to bias, especially because it can exclude many older patients. However, in 2022, most people can now obtain a smartphone at a relatively reasonable price. The COVID-19 pandemic may have further promoted the use of smartphones because quick response (QR) codes became essential for many social activities, including infection-related questionnaires, before entering the hospital. In the current study, only five patients were excluded for lack of access to a smartphone. Moreover, regarding cost-effectiveness, it could be postulated that apps require limited resources and are easily accessible to individuals of all socioeconomic levels, although these issues require further study. Second, the rate of adequate bowel preparation in the control group was lower than 80%, which we used in the sample size calculation. The main reason is that our sample size calculation considered both outpatients and inpatients, whereas we included only outpatients in this study. Outpatient education is time-consuming but ineffective in most centers. Third, the smartphone app did not allow outpatients to ask specific questions, in contrast to traditional in-person consultations. To counter this limitation, we have collected frequently asked questions and written specific, detailed answers. We will add a new section to the app for these questions and answers and update them frequently. Fourth, the split-dose PEG used in this study was the currently recommended regimen, but patients from different countries or regions may have different bowel preparation regimens. Thus, a multilingual app with different bowel preparation regimens for colonoscopy is a desirable tool for the future.

In conclusion, this study demonstrated that our AI-driven smartphone-guided bowel preparation app effectively improved bowel preparation quality in patients scheduled for an outpatient colonoscopy. This AI system may help patients engage in the bowel preparation process and ultimately lead to higher rates of adequate bowel preparation.

## Methods

This study consisted of two parts: the establishment of an AI-driven smartphone app and a clinical trial evaluating the app. The entire study protocol was approved by the institutional review board of Zhongshan Hospital (B2020-297R) and registered at the Chinese Clinical Trial Registry (ChiCTR2000040306). All authors had access to the study data and reviewed and approved the final manuscript.

### Creation of the AI-based bowel preparation prediction system

**Fig. 3:**
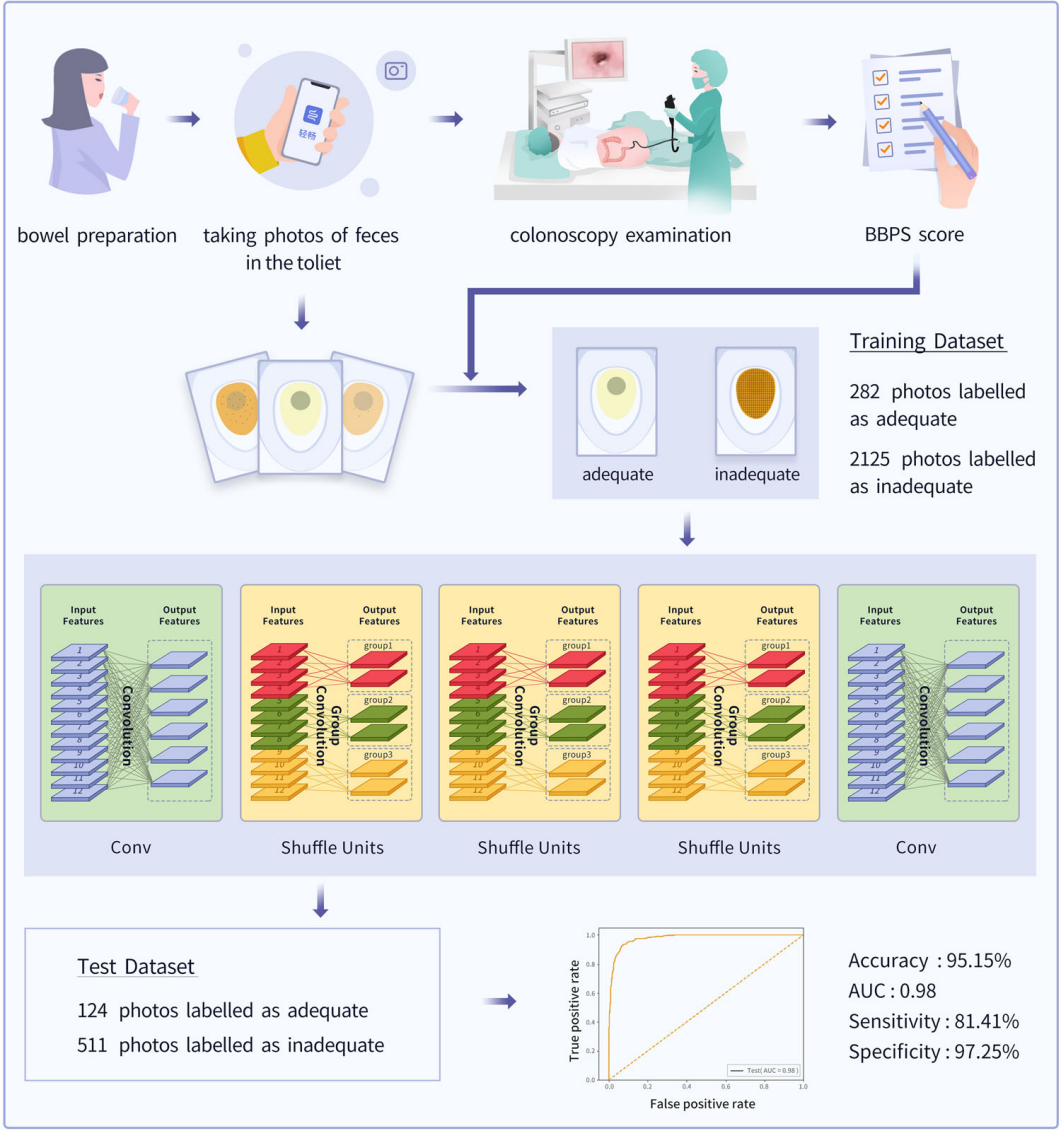
Entire process of creating the artificial intelligence-based bowel preparation prediction system. AUC area under the receiver operating characteristics curve, Conv convolution.

Figure 3 presents the process of creating the AI system. Pictures of feces in the toilet after bowel preparation were used to construct the bowel preparation prediction system. Patients scheduled to undergo colonoscopy were enrolled from November 2020 to January 2021. The 3-L split-dose PEG (Heshuang, Wanhe Pharmaceutical Co, Shenzhen, China) method was used in this part of the study^27^. Patients were asked to take photographs of the residue in the toilet every time they used the toilet after taking all doses of the purgative. The entire colonoscopy procedure was video-recorded, and the BBPS score was determined. Adequate bowel preparation was defined as a total score ≥6 plus all segment scores ≥2 during withdrawal of the colonoscope after cecal intubation, in accordance with the BBPS guide^25,27^. Based on these criteria, the patients were divided into adequate and inadequate bowel preparation groups.

If a patient’s bowel preparation status was inadequate, all their uploaded photographs were labeled as “inadequate.” If their bowel preparation status was considered adequate, the first photograph was labeled as “inadequate,” the last uploaded photograph was labeled as “adequate,” and the intermediate photographs of the series were discarded because the corresponding preparation status could not be confirmed. The photographs were randomly divided at the patient level into a training set (~80% of the images) and a test set (~20% of the images). In total, 5,362 photographs were selected from 992 patients to develop our AI-based bowel preparation prediction method. The data distribution is shown in Supplementary Table 1.

Supplementary Information 1 details the neural network architecture. The training image set was used to train ShuffleNet, and the early stopping strategy was used to avoid over-fitting by monitoring the model’s performance on the internal validation dataset. Image data augmentation, including random cropping and rotation, was performed to increase the generalization and robustness of the model. We evaluated the performance of the classification of the test set by calculating the accuracy, sensitivity, specificity, and AUC.

### Establishment of the AI-driven smartphone app

The AI-based prediction system was integrated into a smartphone app called Qing Chang (version 2.0, provided by Henan Xuanweitang Medical Information Technology Co., China). The design and workflow are detailed in Supplementary Information 2. The app has three main functions. First, it collects patient information, including the patient’s colonoscopy appointment time, the hospital or medical center, and other medical diseases, and uses this information to schedule the entire bowel preparation process. Second, the app delivers personalized reinforced education to the patients, starting prior to the scheduled colonoscopy. The content of the patient education is shown in Supplementary Information 3. Each part contains at least one short video or article focused on improving bowel preparation, all of which were reviewed by two senior endoscopists (Ping-Hong Zhou and Quan-Lin Li). The third function of the app is to evaluate the status of bowel preparation and give the users personalized improvement suggestions based on the AI system’s prediction of bowel preparation quality. Supplementary Fig. 1 presents the schematic overview of the AI-driven app, and Supplementary Fig. 2 shows the workflow of the AI system integrated into the app.

### Clinical trial design

A prospective, multicenter, endoscopist-masked randomized controlled study was conducted between September 2021 and January 2022 at four different endoscopy centers: Zhongshan Hospital, Zhengzhou Central Hospital, Central Hospital of Minhang District, and Xian Central Hospital. The trial protocol is presented in the Supplementary material. The study performance and reporting followed the Consolidated Standards of Reporting Trials, including the use of a flowchart (Fig. 1) to track participants.

### Patient recruitment

Outpatients between 18 and 75 years of age scheduled for routine diagnostic colonoscopy during the study period were eligible for this study. For inclusion in the study, each patient was required to own a smartphone that could access the app.

The exclusion criteria were as follows: (i) previous bowel surgery; (ii) gastroparesis or gastric outlet obstruction; (iii) known or suspected intestinal obstruction or perforation; (iv) severe chronic renal failure (creatinine clearance <30 mL/min); (v) severe congestive heart failure (New York Heart Association class III or IV); (vi) current pregnancy or breastfeeding; (vii) toxic colitis or megacolon; (viii) poorly controlled hypertension (systolic blood pressure >180 mm Hg and/or diastolic blood pressure >100 mm Hg); (ix) moderate or massive active gastrointestinal bleeding (>100 mL/day); (x) major psychiatric illness; (xi) allergy to the study purgatives; (xii) inability to use the smartphone app; or (xiii) inability or unwillingness to provide informed consent.

### Randomization and masking

When scheduling the colonoscopy appointment at the outpatient visit, patients were interviewed by a research assistant not involved in the examination procedures. Written informed consent was obtained from all patients. The assistant explained the aims of the study and collected demographic and medical information on a data collection sheet. The eligible participants were randomized into the control group or the AI-driven app group (i.e., the AI-driven bowel preparation group) in a 1:1 ratio by block randomization with stratification by center. The random allocation table was generated with SAS 9.4 software, and the randomization masking was implemented with an opaque envelope. At least 50 patients were included from each center. Patients were informed of their group assignment and required not to reveal it. None of the attending endoscopists were aware of the patients’ group assignments.

### Education and instructions on the bowel preparation process

When scheduling the colonoscopy during the outpatient visit, all patients were educated about the importance of adequate bowel preparation before the colonoscopy. The standard patient education for bowel preparation included dietary recommendations and information about the use of purgatives, potential adverse drug reactions, and management of inadequate bowel preparation.

All individuals received instructions for a 3-day low-residue diet and a prescription for electrolyte powder (PEG) (Heshuang, Wanhe Pharmaceutical Co). Four liters of PEG were given to each patient during recruitment. Patients began drinking the first liter of PEG at 20:00 on the day before the procedure at a rate of 250 mL every 15 min. On the day of the procedure, patients were instructed to consume 2 L PEG 4–6 h before the examination. The remaining liter of PEG served as a remedial measure for inadequate bowel preparation. Patients enrolled in the control group decided whether this additional 1 L PEG was required using their own judgment. They were told that if their feces were liquid and contained no obvious particles, according to the reference photographs, their bowel preparation was adequate.

Patients in the AI-driven app group were given a link to download the app. The total number of app users and the process of app usage were tracked. The app sent the patients education and reinforcement reminders with suggestions for bowel preparation and indicated whether they should consume the additional 1 L PEG.

### Colonoscopy

All colonoscopy examinations were performed at 8:30–11:30 or 13:30–16:30. Each examination was performed by an endoscopist with a minimum experience of 3000 endoscopic examinations. The insertion goal was to achieve cecal intubation as quickly as possible. The entire colonoscopy procedure was video-recorded. Polyp removal and biopsies were performed during the withdrawal of the scope.

### Outcomes and data collection

The primary outcome was the percentage of patients with adequate bowel preparation, defined as a total BBPS score ≥6 plus all segment scores ≥2. Two endoscopists masked to the entire clinical research reviewed the videos and assigned the BBPS score. If they did not reach a consensus, another senior endoscopist made the final decision.

The secondary outcomes were the total BBPS score, the BBPS score in each colon segment, the rate of patients with perfect bowel preparation (BBPS score ≥8), the rates of compliance with dietary restrictions and purgative instructions, cecal intubation time, colonoscope withdrawal time, PDR, ADR, and aADR. Compliance with dietary restrictions was defined as following the diet instructions and not consuming banned foods. Compliance with purgative instructions was defined as taking the correct volume of purgatives at the correct starting time. PDR was defined as the percentage of patients with ≥1 polyp. ADR was defined as the percentage of patients with ≥1 adenoma. Advanced adenomas were defined as adenomas with an endoscopic size of ≥10 mm, high-grade dysplasia, or villous features.

Patients were also asked to rate their sleep quality during the bowel preparation process as the same as usual or worse than usual and share whether they would be willing to undergo bowel preparation for a repeat colonoscopy in the future.

### Statistical analysis and sample size calculation

The rate of adequate bowel preparation at our endoscopic centers is ~80%. We expected the app to increase this percentage to 90%. To detect this difference with a significance level (α) of 0.05 and a power of 80% using a two-tailed test, we calculated that ~394 patients were required for this study. Considering that ~20% of patients cancel their colonoscopy appointment, we estimated that 500 patients would be required to detect a significant difference in the primary outcome.

The FAS consisted of all participants except those who canceled their colonoscopy appointment after randomization. Patients in the AI-driven app group were expected to take photographs for bowel preparation prediction and browse more than half of the educational content on the app. A research assistant checked and recorded the usage of the app from each patient’s smartphone after the completion of the colonoscopy. Patients who did not use the app in this manner were excluded from the PPS. The SS included all patients who underwent colonoscopy with a safety assessment during the trial. The rates of adequate and excellent bowel preparation were analyzed in the FAS and PPS, and other secondary endpoints were analyzed in the FAS. The safety of the intervention was analyzed in the SS. All statistical analyses were performed with SAS software (version 9.4) at the 0.05 significance level unless otherwise noted. Because the secondary outcomes were considered exploratory, we did not correct the statistical multiplicity generated by multiple outcomes.

Continuous data were presented as the mean ± standard deviation and analyzed using Student’s *t*-test, and categorical data were presented as the number (percentage) and analyzed with the chi-square test or Fisher’s exact test. The rate and its 95% confidence interval (CI) for each group were estimated using the Clopper–Pearson method. The rate difference between the two groups and its 95%CI were calculated using the Newcombe–Wilson method with a continuity correction. The rate of adequate bowel preparation was also compared between the study group and the control group in some subgroups.

### Reporting summary

Further information on research design is available in the Nature Research Reporting Summary linked to this article.

## Supplementary information

Supplemental Material

Reporting Summary

## Data Availability

The data described in this manuscript may be made available upon reasonable request to Ping-Hong Zhou (zhou.pinghong@zs-hospital.sh.cn).

## Code availability

The code may be made available upon reasonable request to Shuo Wang (shuowang@fudan.edu.cn).
