# Supplementary material for: Improving bowel preparation for colonoscopy with a smartphone application driven by artificial intelligence
Source: NPJ Digit Med. 2023 Mar 14;6:41. doi: 10.1038/s41746-023-00786-y (PMC10011797; doi:10.1038/s41746-023-00786-y)
Supplement: Supplementary file 1 — Supplemental Material [file 41746_2023_786_MOESM1_ESM.pdf]

Supplementary Materials

Improving Bowel Preparation for Colonoscopy with a Smartphone Application Driven by Artificial Intelligence

This supplementary material provides additional information about the research. It contains the following items:

1. Development of the AI-based bowel preparation prediction system

2. Workflow of the smartphone application

3. Brief summaries of the patient education content in the Qing Chang app

4. Supplementary figures

5. Supplementary tables

6. Protocol of the clinical trial

Supplementary Information 1: Development of the AI-based bowel preparation prediction system

1. Algorithm design

The prediction of bowel preparation can be formulated as an image classification problem. Let $\mathbf{X} \in \mathbb{R}^{H \times D}$ denote the toilet photograph taken by the camera of the user's smartphone. The task is to predict the preparation status $y \in \{0, 1\}$, where $y = 0$ represents inadequate bowel preparation and $y = 1$ represents adequate bowel preparation. We utilized a convolutional neural network (CNN) $\boldsymbol{f}_{\boldsymbol{\theta}}$ to learn the mapping: $y = \boldsymbol{f}_{\boldsymbol{\theta}}(\mathbf{X})$, where $\boldsymbol{\theta}$ denote the parameters of the CNN. Binary cross entropy (BCE) loss was used as the loss function to optimize the network parameters.

2. Network architecture

Although convolutional neural networks (CNNs) have achieved remarkable performance in the task of natural image classification, the requirement for a high-performance graphics processing unit (GPU) limits their application in the edge computing environment, such as smartphones. In this study, we employed an extremely efficient CNN architecture for image classification called 'ShuffleNet v2', which runs directly on smartphones. 'ShuffleNet v2' is an upgraded version of 'ShuffleNet v1'. It outperforms other lightweight CNNs in terms of accuracy at the same complexity. Compared with the standard convolutional design, the unique channel shuffle mechanism of ShuffleNet (Supplementary Figure 3) allows different groups of convolutions to exchange information so that the input and output are correlated on the channel, while the design of group convolution significantly reduces the computing cost.

Compared to ShuffleNet v1, ShuffleNet v2 redesigned the convolution and optimized the channel split for memory efficiency (Supplementary Figure 4).

ShuffleNet v2 is an open-source framework, and the codes are publicly available for reproducible research. We adopted the backbone of the original ShuffleNet v2 but modified the final fully connected layers to enable binary prediction of bowel preparation status.

3. Datasets

For the binary image classification, we collected photographs uploaded from patients (after obtaining their consent). The patients were asked to upload a photograph of the home toilet bowl after they took the polyethylene glycol electrolyte solution for bowel preparation. For the series of photographs uploaded by each patient, the classification labels were determined retrospectively according to the final bowel preparation status based on the BBPS scores of their colonoscopy videos. If the bowel preparation status was inadequate ($y = 0$), all of the uploaded photographs were labelled 'inadequate'. If the bowel preparation status was considered adequate ($y = 1$), the first photograph was labelled 'inadequate' and the last uploaded photograph was labelled 'adequate', while the intermediate photographs in the series were discarded, as the status could not be confirmed.

All photographs were randomly divided at the patient level into a training set (794 patients; approximately 80% of the photographs) and test set (198 patients; approximately 20% of the photographs). In total, we collected 5,362 photographs from 992 patients for the development of AI-based bowel preparation prediction. Of the 992 patients, 636 were found to have adequate bowel preparation at colonoscopy. Data distribution of the training and test datasets is summarized in Supplementary Table 1.

4. Implementation details

We built ShuffleNet with PyTorch 1.2 on Ubuntu 18.04 LTS. Training and experiments were performed on a Linux machine with the following configuration: Intel Core i7-6700K CPU 4.0 GHz processor, 32 GB of DDR3 RAM, Toshiba 1 TB HDD, and NVIDIA GTX1060 on GPU with 6 GB memory. The training dataset was further divided into an internal training set (approximately 80% of the photographs) and an internal validation set (approximately 20% of the photographs) for hyperparameter optimization. The internal training set was used to train the ShuffleNet, and the early stopping strategy was used to avoid over-fitting by monitoring the model performance on the internal validation dataset. Image data augmentation, including random cropping and rotation, was performed to increase the generalizability and robustness of the model.

5. Validation results

After 350 epochs of training, the model converged well and showed satisfactory performance on the test dataset. The classification performance is detailed in Supplementary Table 2.

Supplementary Information 2: Workflow of the smartphone application

In the below workflow outline, we used the following formatting conventions:

- [text] refers to the popup message in the app
- text refers to the time when the message pops up
- (text) refers to the judgment message in the app, which will not be shown to the patient
- {text} refer to changeable content, which will be changed based on the patient's information
- Text refers to the timing of the reminder or judgement message

Reminder messages and the operation process

It requires notification, location, and camera calling permissions; please make sure the phone camera functions normally while in use.

After the app is installed: [(Filling out basic information) Please fill in your time of examination, the hospital of examination, history of chronic disease and other diseases, and history of operations.]

After filling out basic information: [Your time of examination is {examination time}. Please re-confirm your time of examination and make proper travel arrangements to avoid being late.]

[For your safety, please do not drive any motor vehicle within 12 hours after the examination.]

After filling out basic information: [For the quality of your examination, please make proper arrangements with the hospital about your time of examination if you have an abdominal and pelvic ultrasound, gastrointestinal imaging, or other tests on the same day.]

After filling out basic information: Play the video "Indications and contraindications of colonoscopy examination"

(Based on the information of chronic disease filled out by the patient) Play the video and educational material "Precautions for patients with chronic disease"

8:00, 3 days before the examination: Play the video and educational material "Recommended diet for three days before the examination"

[Please refer to the recommended diet plan and arrange your meals; this is very important for your bowel preparation]

8:00, 3 days before the examination: Play the video and educational material “Significance of colorectal cancer screening”

8:00, 2 days before the examination: Play the video and educational material “The diet before colonoscopy”

[Please refer to the recommended diet plan and arrange your meals; this is very important for your bowel preparation]

8:00, 2 days before the examination: Play the video and educational material “Precautions in colonoscopy”

8:00, 1 day before the examination: Play the video and educational material “The diet before colonoscopy”

[Please refer to the recommended diet plan and arrange your meals; this is very important for your bowel preparation]

[Please choose a liquid diet of few dregs tonight]

17:00, 1 day before the examination: [Please do not have anything to eat after dinner tonight]

19:50, 1 day before the examination: Play the video and educational material “How to prepare the purgatives”

[Start taking medicine at 8:00 p.m.; please make 1 L of PEG in advance]

20:00, 1 day before the examination: [Medication time, please take your medication on time]

20:00, 1 day before the examination: Play the video “Possible unpleasant effects during medication”

App starts counting steps of the patient [Please walk around a bit after taking the medication and take your mobile phone with you]

20:30, 1 day before the examination: (If the step count is less than 100 from 20:00 to 20:30) [Please

walk around more and rub your belly gently]

<u>21:00, 1 day before the examination</u>: [Please set the alarm for tomorrow at {6 hours and 30 minutes before the scheduled examination} to remind you to take the medicine on time]

<u>21:00, 1 day before the examination</u>: (If the step count is less than 100 in the previous 30 minutes) [Please walk around more and rub your belly gently]

<u>22:00, 1 day before the examination</u>: [Please go to bed early]

<u>6 hours and 10 minutes before the examination</u>: [Take your medicine at {6 hours before the examination}; please prepare 2 L of PEG in advance]

<u>6 hours before the examination</u>: [Take laxatives; please finish in 60–90 min]

<u>6 hours before the examination</u>: Play the video “Management of inadequate bowel preparation”

<u>5 hours and 30 minutes before the examination</u>: (If the step count is less than 100 in the previous 30 minutes) [Please walk around more and gently rub your belly]

<u>5 hours and 30 minutes before the examination</u>: [From now on, please take a picture of your feces in the toilet using the app after every time of defecation for prediction of bowel preparation]

<u>5 hours before the examination</u>: (If the step count is less than 100 in the previous 30 minutes) [Please walk around more and gently rub your belly]

<u>4 hours and 30 minutes before the examination</u>: (If the step count is less than 100 in the previous 30 minutes) [Please walk around more and gently rub your belly]

<u>4 hours before the examination</u>: (If the step count is less than 100 in the previous 30 minutes) [Please walk around more and gently rub your belly]

3 hours and 30 minutes before the examination: (If the step count is less than 100 in the previous 30 minutes) [Please walk around more and gently rub your belly]

3 hours before the examination: (If the step count is less than 100 in the previous 30 minutes) [Please walk around more and gently rub your belly]

(Bowel preparation predicted as unqualified based on the pictures of feces in the toilet) [Your bowel preparation is not adequate; please continue]

Play the video “Management of inadequate bowel preparation”

(Bowel preparation predicted as qualified based on the pictures of feces in the toilet) [Bowel preparation completed]

(The time is 3 hours before the examination, and the patient failed bowel preparation three times or more) [You may take another 1 L of PEG]

2 hours and 30 minutes before the examination: (If the step count is less than 100 in the previous 30 minutes) [Please walk around more and gently rub your belly]

2 hours before the examination: [It’s almost your examination time; please leave for the hospital on time]

30 minutes before the examination: [Notice: examination to start in half an hour]

1 hour after the examination: [Please confirm whether the colonoscopy was completed on time]

1 hour after the examination: Play the video “Notice after colonoscopy”

Supplementary Information 3: Brief Summaries of the Patient Education Content in the Qing Chang App

1. Indications and contraindications for colonoscopy examination

Brief summary: Before colonoscopy examination, patients should be aware of the indications and contraindications of the procedure. This part details all of the contraindications for colonoscopy, including the relative contraindications.

2. Dietary suggestions and restrictions

Brief summary: In this part, a 4-day dietary program is introduced. The patients will receive dietary suggestions and information on dietary restrictions every day at 8:00 a.m. The recommendations will differ according to the amount of time remaining before the examination: 2–3 days before the examination, the day before the examination, and the day of the examination.

3. How to prepare the purgatives

Brief summary: This part mainly introduces the preparation and dosage schedule of the purgatives. The 4-L polyethylene glycol (PEG)-based spilt-dose regimen was used.

4. Adverse events and management

Brief summary: This part introduces potential adverse reactions related to bowel preparation, including flatulence, vomiting, nausea, headache, dizziness, palpitations, and abdominal pain, as well as their therapeutic measures.

5. Precautions for patients with chronic disease

Brief summary: This part introduces some matters requiring attention in patients with chronic diseases, including hypertension, diabetes, asthma, and bronchitis.

6. Management of inadequate bowel preparation

Brief summary: This part introduces measures to improve bowel preparation.

7. Importance of screening for colorectal cancer by colonoscopy examinations

Brief summary: The importance of periodic colonoscopy examinations and other screening tests are introduced in this part.

8. Matters requiring attention after the colonoscopy examination

Brief summary: This part contains suggestions for patients who just underwent colonoscopy, including driving issues, dietary advice, and potential subsequent therapy after pathologic examination of biopsy specimens.

Supplementary Figures

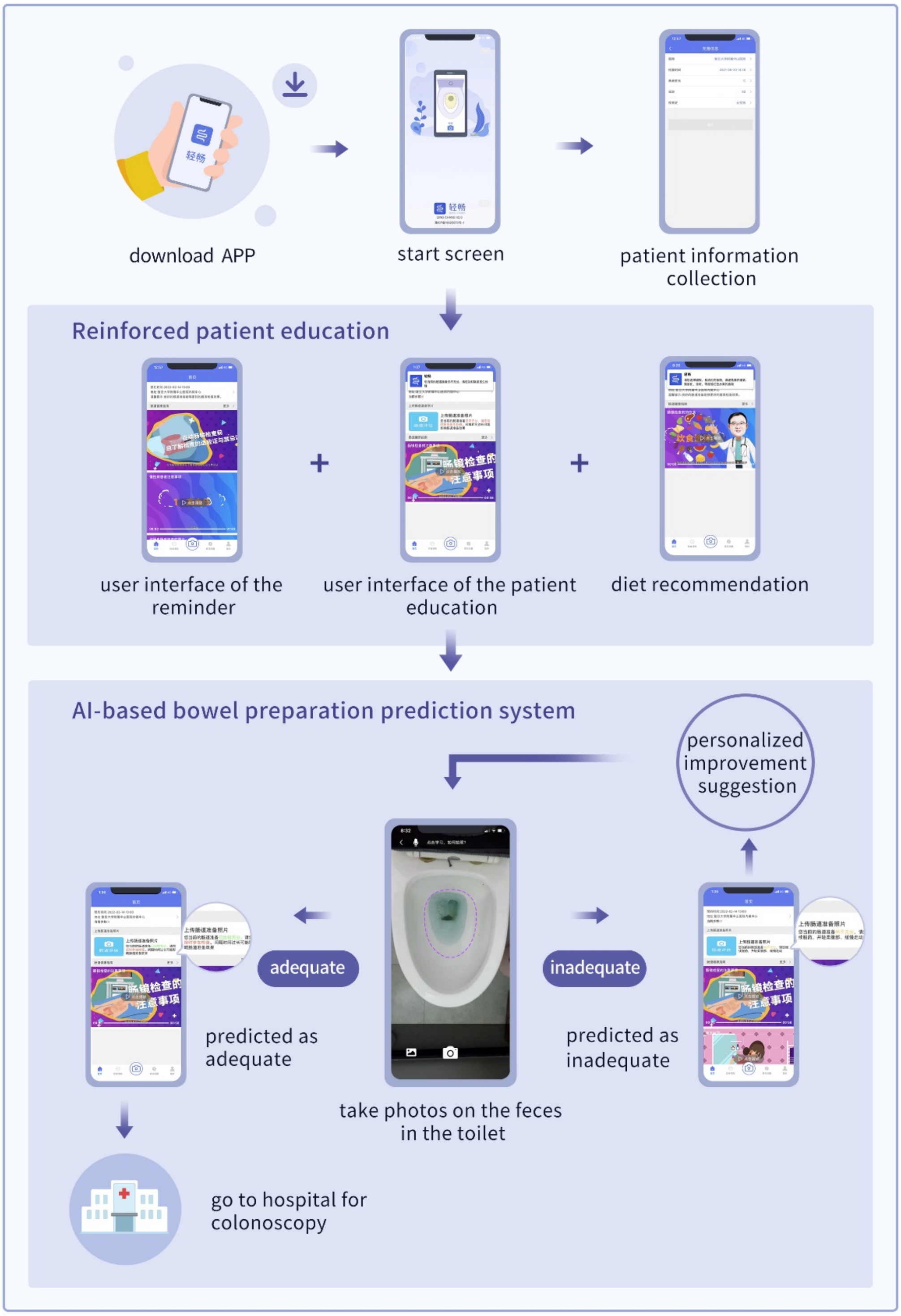

Supplementary Figure 1. Schematic overview of the artificial intelligence–driven smartphone application. App, application.

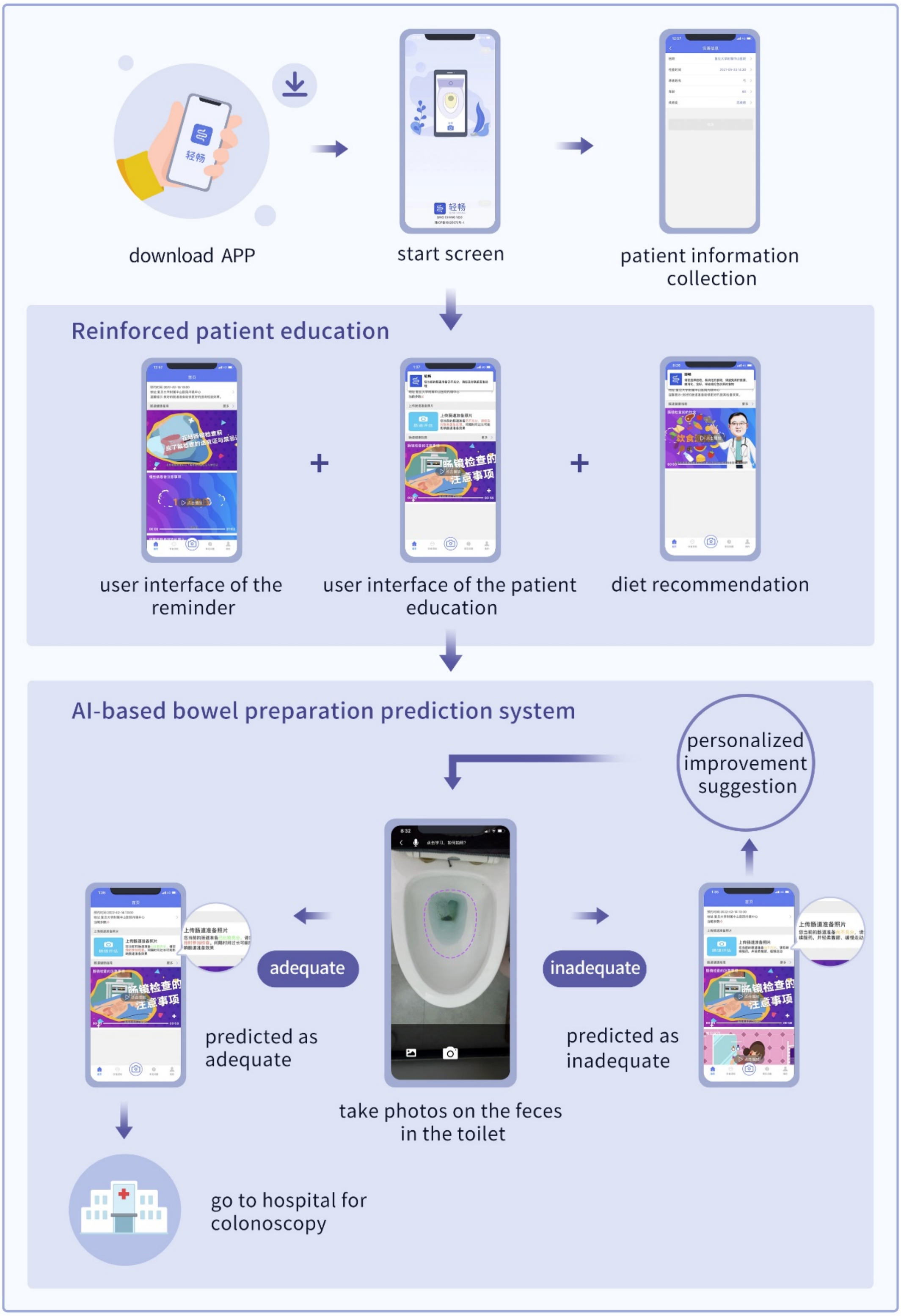

Supplementary Figure 2. Workflow of the artificial intelligence–based bowel preparation prediction system. App, application; Conv, convolution; FC, fully connected.

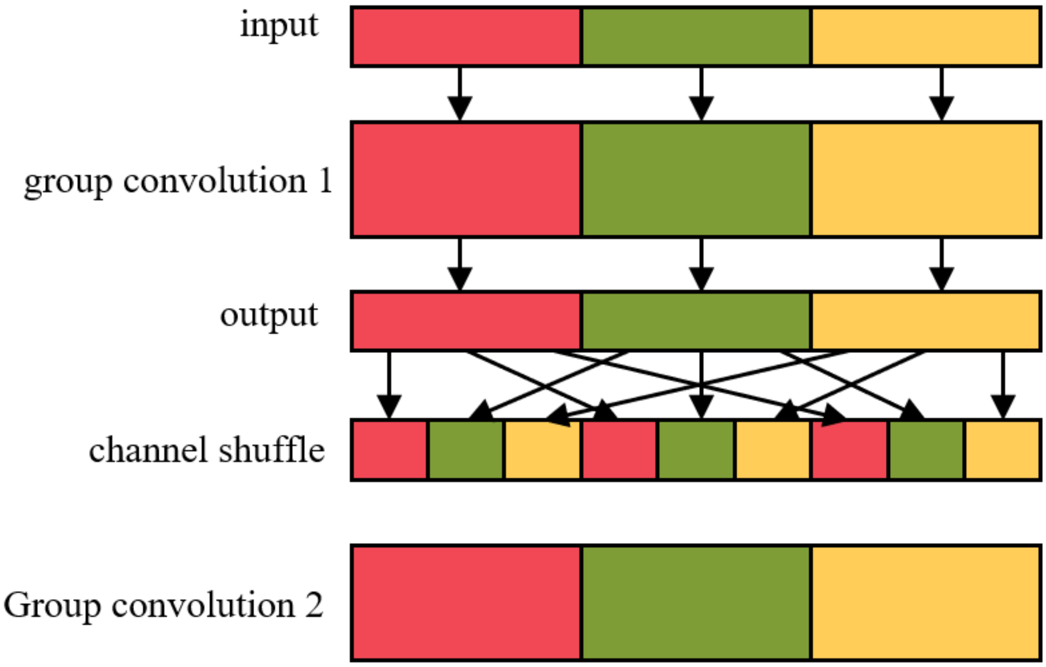

Supplementary Figure 3. Channel shuffle mechanism in group convolutions.

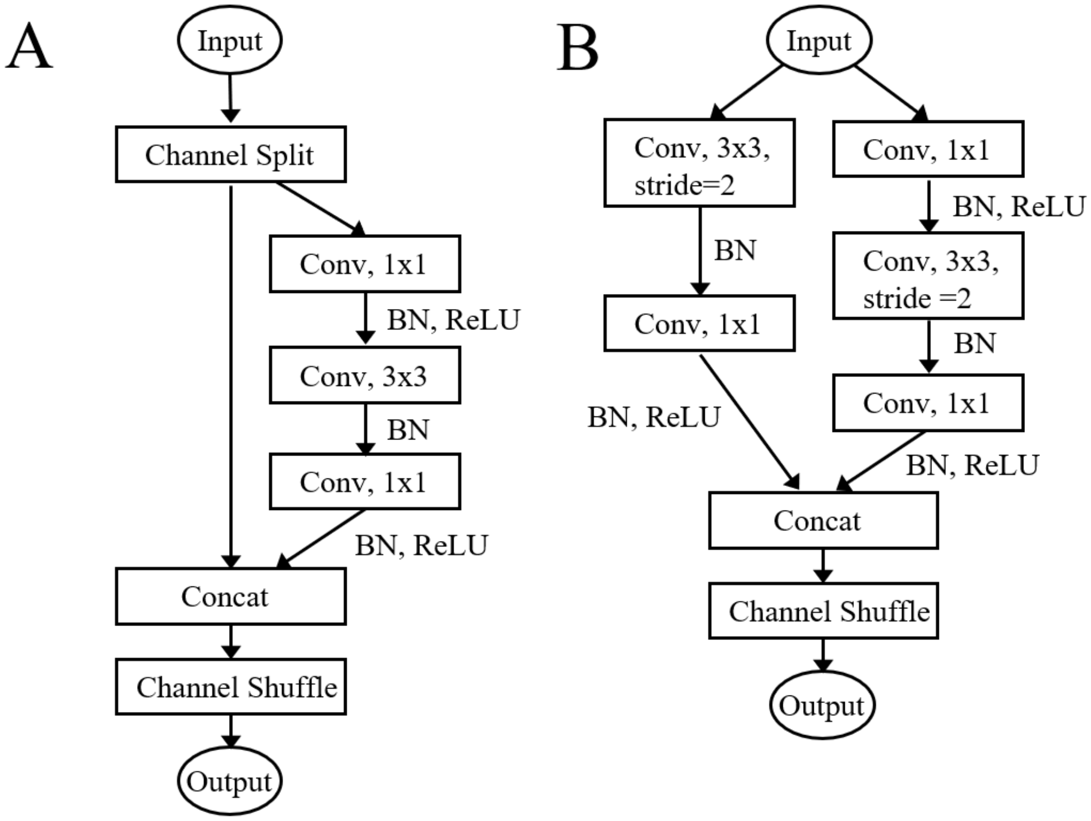

Supplementary Figure 4. A. Basic units of ShuffleNet. B. Down-sampling unit of ShuffleNet. BN, batch normalization; Concat, concatenation; Conv, convolution; DWConv, depth-wise convolution; ReLU, rectified linear unit.

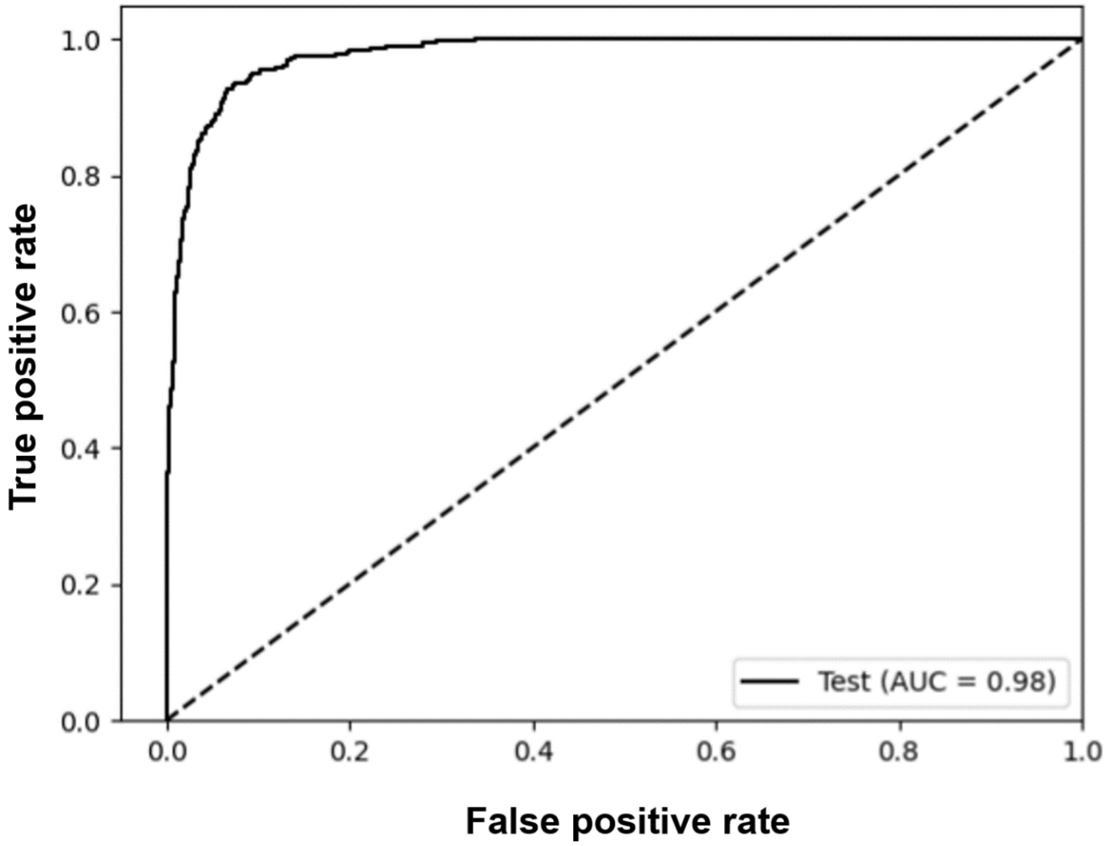

Supplementary Figure 5. Receiver operating characteristics curve of the artificial intelligence–driven smartphone application for predicting adequate bowel preparation. AUC, area under the curve.

Supplementary Tables

Supplementary Table 1. Overview of the image datasets for training and testing

Patients and Photographs	Training Dataset (n)	Test Dataset (n)
Total number of patients	794	198
Patients with adequate bowel preparation	512	124
Patients with inadequate bowel preparation	282	74
Total number of photographs	4,322	1,040
Photographs designated as adequate	512	124
Photographs designated as inadequate	2,006	511
Discarded photographs (unknown status)	1,804	405

Supplementary Table 2. Performance of binary classification on the test dataset

Sensitivity	Specificity	Accuracy	AUC
81·41%	97·25%	95·15%	0·98

AUC, area under the receiver operating characteristics curve.

Study Protocol

1. Summary

Trial design	Prospective, multi-center, endoscopist-blinded, randomized, controlled study
Experimental group	Receiving reinforced education from the AI-driven app
Control group	Receiving standard education for bowel preparation
Primary endpoint	The rate of patients with adequate bowel preparation
Secondary endpoints	BBPS score; rate of patients with perfect bowel preparation; compliance with instructions; polyp detection rate (PDR); adenoma detection rate (ADR); advanced adenoma detection rate (aADR); mean polyps per patient (MPP); mean adenomas per patient (MAP)
Participants	Outpatients scheduled to undergo colonoscopy examination in the endoscopy center
Sample size	500 in total

2. Study background

The recent global estimates of cancer incidence and mortality place colorectal cancer (CRC) as the fourth most prevalent and second deadliest cancer worldwide[1]. The combination of a well-defined precursor lesion and a long preclinical course make CRC an ideal candidate for cancer prevention screening[2]. Colonoscopy is regarded as the principal method for CRC screening; however, it might be affected by many factors, among which the quality of bowel preparation is one of the most important predictors[3]. The optimal bowel preparation is the prerequisite of a successful colonoscopy, including appropriate volume purgatives, accurate schedule reminder, and at least 3-day diet restrictions[4,5]. Consequently, the incidence of inadequate bowel preparation was up to 20%–25%[6,7] according to the existing research. Patients with inadequate bowel preparation are unwilling to follow preparation instructions, struggle with the prescribed diet, and are unable to tolerate the full course of purgative[8]. Therefore, it is necessary to provide reinforced education on bowel preparation to patients.

The reinforced education of bowel preparation can be divided into two approaches, visual aids and reminder functions. The visual aids methods include patient educational booklets[9], cartoon visual aids[10], and nurse-delivered education with brochures[11]. Meanwhile, short messages[12], telephone-based-instructions[13], and smartphone applications (apps)[14,15] can serve as reminders to give instructions on bowel preparation. The WeChat (the most widely used social media platform in China) might be a good solution for the combination of visual aid and reminder messages[16], but it is still limited by poor user experience or lacking engagement with the patient.

These existing approaches have proved effective but have the following problems. The most important issue is that patients cannot evaluate whether their bowel preparation is adequate. The visual aid such as photographs of ‘clean’ and ‘dirty’ feces in the toilet might be useful, but it cannot cover all kinds of situation and was hard for the elder patients to identify. Besides, it is hard for elder patients or patients with different educational background to comprehend long paragraphs of text or dialogue. Furthermore, the reminder function may fix all the schedule without considering the different status of bowel preparation of different patients. Patients should receive personalized remind messages referring to their current bowel preparation (adequate or inadequate).

In recent years, within the field of endoscopy, artificial intelligence (AI) is expected to have the potential to overcome certain clinical-human hurdles and achieve great improvements[17-19]. Our preliminary experiments revealed the AI system can be applied to evaluate the bowel preparation status according to the feces in the toilet. This reminds us that the development of an AI-driven smartphone guided bowel preparation method might provide more personalized and accurate enhanced instructions to improve patients’ bowel preparation. The AI system acts as an evaluator, while the smartphone App acts as a reminder according to the evaluation results of the AI system.

In this research, we intended to construct an AI-based bowel preparation prediction system for the outpatient who will undergo colonoscopy examination. Such prediction system can predict the quality of bowel preparation in real-time through assessing the feces in the toilet. An AI-driven smartphone app will be established based on the AI-based bowel preparation prediction system and provide personalized enhanced instructions to improve patients’ bowel preparation. The aim of the prospective multicenter study will be to evaluate the impact of this AI-driven smartphone guided bowel preparation method on the quality of bowel preparation in outpatients undergoing colonoscopy.

3. Flow chart

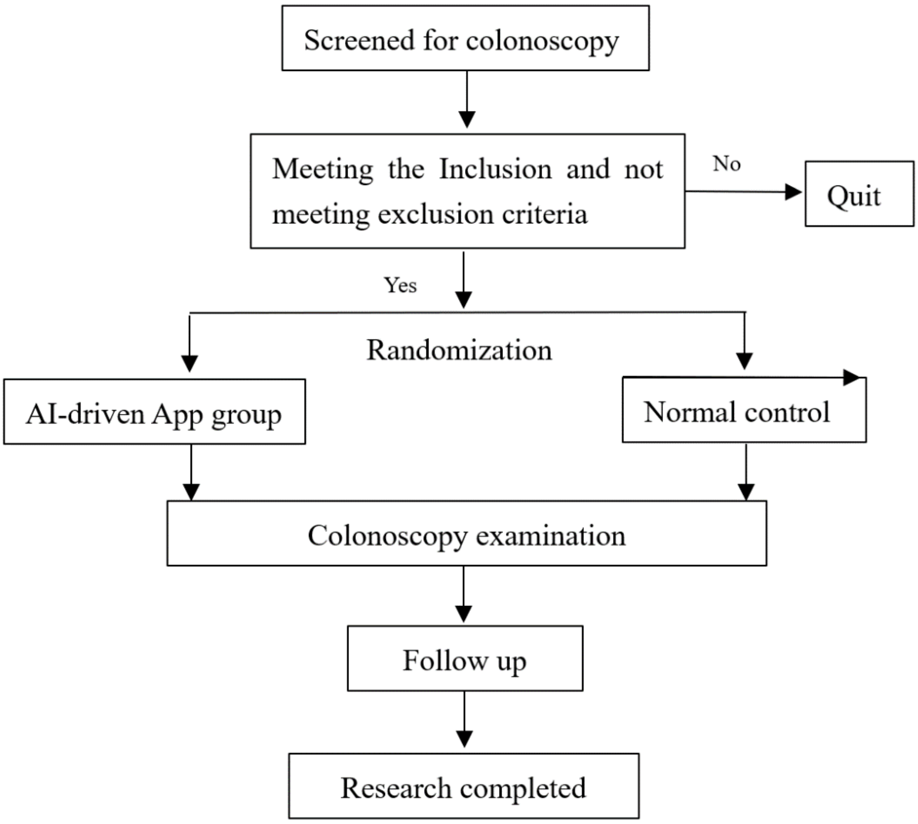
Screened for colonoscopy
Meeting the Inclusion and not meeting exclusion criteria
No
Quit
Yes
Randomization
AI-driven App group
Normal control
Colonoscopy examination
Follow up
Research completed

4. Patient selection

4.1 Inclusion criteria

(1) Outpatients between 18 and 75 years old who are receiving routine diagnostic colonoscopy.

(2) The patients should have a smartphone to access the application.

4.2 Exclusion criteria

Patients meeting these exclusion criteria will be excluded: (1) former history of bowel surgery; (2) gastroparesis or gastric outlet obstruction; (3) known or suspected intestinal obstruction or perforation; (4) severe chronic renal failure (creatinine clearance <30 mL/min); (5) severe congestive heart failure (New York Heart Association class III or IV); (6) pregnancy or lactation; (7) diagnosis of toxic colitis or megacolon; (8) poorly controlled hypertension (arterial systolic blood pressure >180 mm Hg and/or arterial diastolic blood pressure >100 mm Hg); (9) moderate or massive active GI bleeding (>100 mL/day); (10) major psychiatric illness; (11) allergy to purgatives; (12) inability to use a smart phone application; or (13) unable to give informed consent or unwilling to participate.

4.3 Definition of enrollment

After participants signed informed consent and being randomized, they will be regarded as enrolled. The randomization time will be the enrollment time and recorded in the CRF.

5. Endpoints

5.1 Primary endpoint

The primary endpoint is the rate of patients with adequate bowel preparation. During the past decade, the Boston Bowel Preparation Scale (BBPS) has been applied in practice and regarded as the standard evaluation scale. Each segment of the colon (proximal, transverse and distal colon) will be independently assessed providing a BBPS score from 0 to 3 points. The adequate bowel preparation will be defined as a total score ≥6 with all segment scores ≥2 during withdrawal of the colonoscope after cecal intubation, according to the guide and former high-quality research. Each of the 3 segments of the colon (right, including the cecum and ascending colon; transverse, including the hepatic and splenic flexures; and left, including the descending colon, sigmoid colon, and rectum) was assigned a score from 0 to 3, defined as follows: 0, unprepared colon segment with the mucosa barely seen because of solid stool that cannot be cleared; 1, portion of the mucosa of the colon segment seen but other areas of the colon segment not seen well because of staining, residual stool, or opaque liquid; 2, minor amount of residual staining, small fragments of stool, and/or opaque liquid, but mucosa of the colon segment seen well; 3, entire mucosa of the colon segment seen well with no residual staining, small fragments of stool, or opaque liquid. To minimize interobserver variability, pre-enrollment training was given to optimize scoring accuracy. Before the study started, three endoscopists were asked to review colonoscopy videos of 30 patients and

evaluate the bowel preparation effectiveness using BBPS. Afterwards, they discussed the BBPS score of each patient and reached a consensus. In this research, two senior endoscopists who are blinding to the whole research will review the colonoscopy examination videos and give the BBPS. If they cannot reach the consensus, another senior endoscopist will give the final score without knowing the former evaluation results.

5.2 Secondary endpoints

The secondary endpoints include the BBPS score, BBPS score in different regions, the rate of patients with perfect bowel preparation (BBPS score ≥8), compliance with diet restrictions and purgative instructions, cecal intubation time, withdrawal time, polyp detection rate (PDR), adenoma detection rate (ADR), and advanced adenoma detection rate (aADR).

The PDR is defined as the percentage of patients undergoing colonoscopy who have one or more polyps. The ADR is defined as the percentage of patients undergoing colonoscopy who have one or more adenomas. Advanced adenomas are defined as adenomas with an endoscopic size ≥10 mm, high-grade dysplasia, or villous features. Compliance with diet restrictions is defined as following the diet instructions without taking banned food. Compliance with purgative instruction is defined as correct starting time and volume for taking purgatives. Sleeping quality is defined as same as usual or worse than usual.

6. Purpose

The aim of the prospective multicenter study is to evaluate the impact of this AI-driven smartphone guided bowel preparation method on the quality of bowel preparation in outpatients undergoing colonoscopy.

7. Study process

7.1 Enrollment

Only after patients signed the informed consent, can the research-related procedures be conducted.

7.2 Informed consent

According to the Helsinki Declaration, patients are not allowed to participate in the study without adequate informed consent. The principal investigator is responsible for ensuring that no patient was enrolled in the study without adequate informed consent. Failure to obtain informed consent and failure to document this process is considered a violation of the Helsinki Declaration and the study protocol. All informed consent documents (ICDs) must be approved by the ethics committee. Patients' informed consent requires documentary record on the informed consent by themselves in their primary language.

The investigator or trained designated person performs a preliminary screening to determine if the patient generally meets the eligibility criteria for the study. If yes, the investigator or trained designee should

recommend the patient to participate in the study. If the patient agrees to participate, they will need to sign an informed consent document.

The investigator or trained designated person should confirm that the subject understands the following points in the study:

• the purpose of the research,

• potential risks or adverse events,

• potential risks or adverse events directly related to participating the research,

• the likelihood of failure,

• research requirements include follow-up visits, and

• all rights of the subject as a participant in the clinical study.

After explaining the purpose of the study, the investigator or trained designee should answer any questions from the subject. If the subject agrees to participate, his or her wishes must be recorded by signing and dating the ICF, and the document should be signed and dated by the patient providing the informed consent. After successful completion of the informed consent process, the investigator or trained designated person will assess the eligibility of patients based on the protocol.

7.3 Patient selection

Outpatients who undergo colonoscopy examination and generally meet the study requirements will be screened based on the inclusion/exclusion criteria. Patients who pass the screening will be enrolled and recorded in the subject screening and enrollment tables. There will be no bias in the choice of enrolled subjects. The date of screening, the results (enrolled or not), and the primary reason for not selecting subjects (such as not meeting inclusion criteria or not interested in participating in the study) will be recorded. After the patients are enrolled, the research center should complete the preoperative study data collection. It is desirable to be able to collect complete data for all enrolled patients, without those withdrawn from the study.

7.4 Subject identification number

Patients will be numbered after signing informed consent. The subject number begins with QC as a fixed number and is numbered starting from 0001 at the time of signing the informed consent. For example, the first patient who signs the informed consent will be QC001, and the second one will be QC002. Once the subject identification number is assigned, the number is not reusable.

7.5 Treatment description

This section applies to individuals who have signed an informed consent form and have been identified as eligible to participate in this study on the basis of the inclusion and exclusion criteria. This section introduces the whole process of the colonoscopy (including the bowel preparation, examination and data

collection) in detail.

7.6 Bowel preparation management

During the outpatient visit, all patients will receive patient education on the importance of adequate bowel preparation before colonoscopy. The standard instructions of bowel preparation include dietary recommendation, usage of the purgatives, adverse drug reactions, and management of inadequate bowel preparation. Instructions for a 3-day low-residue diet are given. Electrolyte powder (PEG) (HeShuang, WanHe Pharmaceutical Co, Shenzhen, China) is prescribed to each person. The 4-L PEG-based split-dose method will be applied in this research. Patients begin drinking the first 1 L of PEG at 20:00 p.m. on the day before the procedure at a rate of 250 mL every 15 minutes. On the day of the procedure, patients will be instructed to take the 2 L of PEG at 4–6 hours before the examination. The remaining 1 L of PEG will serve as a remedial measure for inadequate bowel preparation. All of the colonoscopy examinations will be performed at 8:30–11:30 or 13:30–16:30.

The patients enrolled in the control group will choose whether the additional 1 L PEG is needed according to their own judgement. The liquid feces with no clear particles awere recommended as adequate bowel preparation.

The patients enrolled in the AI-driven App group will be provided a link or QR code to access the website to download the App. The access to the AI-driven App will be limited to those patients who passed validation. The total number of the application user and the process of the usage of application will be tracked. The patients will receive education and reinforcement reminders on the suggestions on bowel preparation. They should choose to use the additional 1L PEG according to the suggestion of the App.

7.7 Colonoscopy managements

All the colonoscopy examination will be performed by endoscopists with a minimum experience of 3000 endoscopic examination. During the examination, sedation will be performed using propofol. The insertion goal is to achieve cecal intubation as quickly as possible. The whole procedure of the colonoscopy will be recorded. Polyps removal and biopsies were only performed during withdrawal.

7.8 Post-procedure managements

Patients will receive postoperative routine nursing. The study endpoints, complications will be recorded.

7.9 Suspension and withdrawal

Patients who were screened and confirmed to be eligible for the study, signed the informed consent and completed the randomization were considered as enrolled. If serious program deviation, withdrawal, or death occurs, the subject study is considered to be suspended. If the subject

discontinues the study after obtaining informed consent, the data before the discontinuation will still be included in the study-related analysis.

7.9.1 Pre-procedure

In any time during the study period, even before colonoscopy, participants could withdraw their informed consent whenever necessary. Researchers can withdraw participants before surgery according to safety considerations in the inclusion and exclusion criteria.

7.9.2 Intraprocedure

For safety reasons, the investigator may have the subject quit during the procedure. For example, the patient is not suitable for receiving the instrument for the study or the endoscopists do not use the specified instrument for any reason. If the following serious cases occur, please withdraw during the operation:

1） Perforation

2） Bleeding

3） Allergy to narcotic drugs

7.9.3 Replacement

Subject will be deemed to have commenced the study upon completion of the informed consent process, and any subject who has been discontinued prior to or during the endoscopic examination will not subsequently be replaced by other subjects.

7.10 Randomization

The eligible participants will be randomized into the control group or the AI–driven app group (i.e., AI-driven bowel preparation group) in a 1:1 ratio by block randomization with stratification by center. The random allocation table was generated by SAS 9.4 software, and the randomization masking was implemented by opaque envelope. The randomization time (accurate to minutes), randomization person, and subject number and acronym will be recorded.

7.11 Blinding implementation and protection

Blinding method will be adopted for patients and evaluators according to the study protocol. During and after the examination, researchers and the endoscopists performing the colonoscopy should pay attention to avoid discussing random content with the patients and avoid unnecessary unblinding. Patients will be informed of their grouping, but they will not be aware of the influence caused by the different methods of education and will be required to not reveal their group to any endoscopists. All attending endoscopists and assistant nurses will be blinded to the patients' randomization and grouping situation.

7.12 Unblinding

Uncovering or breaking the blinding means displaying the randomized results to the subjects or evaluators. Researchers should protect the blinding method as much as possible. Uncovering the randomization results of a single subject may lead to the leakage of the randomization results of other subjects. Any leakage of the randomization results will have a significant impact on the statistical analysis. In general, it is prudent to deal with the disclosure of blinding law. In the following cases, it is possible to consider the disclosure of blinding law:

1) Endangering the safety of subjects: For example, in some serious adverse events, subjects need to know the randomized results to inform other physicians to take appropriate emergency treatment.

2) Threatening the safety of the assessor: When the assessor is facing potential safety hazards, he or she needs to know the randomization results.

3) Compliance: For instruments and cases reasons of partial compliance, in certain circumstances, if the research involved in unexpected adverse events are needed, randomization should be made public to the relevant departments or the public.

4) Other management reasons.

7.13 Concomitant therapy and medications

The concomitant treatment and concomitant medications will be recorded from the time the subject signs the informed consent to the time the study is completed. Concomitant treatment and concomitant medications should be recorded in the CRF, or a clear copy of the form available from the research center should be kept in the CRF as research data to identify other factors affecting the end of the study. When recording the concomitant treatment and concomitant medications, the indications and the starting and ending time of use should be clearly defined, and the corresponding types of adverse events should be indicated for the treatment measures to cope with adverse events. When using a copy of the study center form, the investigator should sign the copy and indicate the date of review to confirm that the document is a study document.

8. Basis of study protocol and risk/benefit analysis

8.1 Basis of overall design

This study is a prospective, single-blind, randomized, parallel-design, multicenter study.

Prospective: In order to reduce the deviation caused by various factors and avoid the subjective influence on the research results in retrospective studies, the research is carried out under the predetermined test purposes and test methods in order to reach more objective conclusions.

Parallel control design: Subjects will be divided into a control group and an AI-driven app group. The effectiveness and safety of methods used in the two groups will be compared to increase the operability and reliability of the experiment.

Randomization: Subjects will be randomly divided into two groups to avoid bias of the level of physician

diagnosis and treatment on the results of diagnosis and treatment.

Blinding: The endoscopists who perform the colonoscopy examination will be blinded. The evaluator for the BBPS score or pathologist, as the evaluator of the images, will accept the evaluator's blinding method. Patients will be informed of their grouping, but they will not be aware of the influence caused by different methods of education and will be required to not reveal the grouping to any endoscopists.

8.2 Selection of endpoints

The primary endpoint is the rate of patients with adequate or inadequate bowel preparation.

The secondary endpoints include the BBPS score, BBPS score in different regions, percentage of patients with perfect bowel preparation (BBPS score ≥8), compliance with diet restrictions and purgative instructions, cecal intubation time, withdrawal time, polyp detection rate (PDR), adenoma detection rate (ADR), and advanced adenoma detection rate (aADR). Patients' subjective feelings regarding preparation with the app will be assessed using a numeric rating scale (NRS).

8.3 Adverse events

The relevant definitions of adverse events are as follows:

Adverse events (AE): Any adverse medical event, unexpected disease or injury, or adverse clinical manifestations (including abnormal laboratory findings) that occur in a subject, user, or other person, whether or not associated with medical devices.

Serious adverse events (SAE): Adverse events with the following information:

- Causing death
- Leading to severe deterioration of the health of the subjects, including leading to life-threatening diseases or injuries
- Causing impairment of body structure or function
- Resulting in hospitalization or extended hospitalization (leading to hospitalization and preventive medical or surgical intervention)
- Causing permanent damage to body structure(s) or function
- Leading to fetal distress, fetal death, or congenital abnormalities or congenital defects

Note: Hospitalization for existing conditions or surgery required in the program, without serious deterioration of health status, is not considered a serious adverse event. Purposeful hospitalization, such as for economic or reimbursement reasons, is not considered a serious adverse event.

Unexpected adverse device response (UADE): This refers to adverse events related to medical devices that were not previously identified in the current version of the risk analysis report in terms of nature, severity, or incidence. The definition includes any event caused by insufficient or inadequate description of the use or deployment of the device. This definition includes any event caused by a user's error.

8.4 Expected and trial-related adverse events

Previous studies have shown that the expected adverse events are basically the same as the complications of conventional endoscopic diagnosis and treatment.

The instrument used in this experiment is a medical software that is not in contact with the human body. There is no difference between the experimental operation and the routine operation. The intervention measures (such as randomization process) in the experiment may slightly increase the quality of bowel preparation.

8.5 Risk minimization

In this study, when the patients cannot access the smartphone app, they can still apply the normal routine of bowel preparation, greatly reducing the risk of the test.

8.6 Related benefits

The smartphone application may improve the quality of bowel preparation in colonoscopy examination.

8.7 Overall feasibility analysis

Several previous studies proved that reinforcing patient education with a smartphone app optimized bowel preparation in the 3 days before colonoscopy, increasing bowel cleanliness, adenoma detection, and compliance in patients undergoing CRC screening or surveillance. The overall feasibility is strong.

9. Statistical analysis

9.1 Statistical analysis plan

Data management and statistical analysis will be implemented by Zhongshan Hospital.

9.2 Methods

The full analysis set (FAS) consisted of all participants except those who cancelled their colonoscopy appointment after randomization. Patients in the AI–driven app group will be expected to take photographs for bowel preparation prediction and browse more than half of the education content on the app. Patients who did not use the app in this manner will be excluded from the per-protocol set (PPS). Safety set (SS) includes all patients who receive colonoscopy with safety assessment during the trial. The rate of adequate and excellent bowel preparation will be analyzed in the FAS and PPS, and other secondary endpoints will be analyzed in the FAS. The safety of the intervention will be analyzed in the SS. All statistical analyses will be performed with SAS software (version 9.4), and tests wwill be done at the 0.05 significance level unless otherwise noted.

Continuous data will be presented as the mean ± standard deviation (SD) and analyzed using student t-test, while categorical data will be presented as numbers (percentages) and analyzed by chi-square test or Fisher exact test. The rate and its 95% confidence interval (CI) for each group will be estimated by Clopper-Pearson method. The rate difference between two groups and its 95%CI will be calculated using Newcombe-Wilson method with a continuity correction. The rate of adequate bowel preparation will also compared between the study group and control group in some subgroups such as sex, age (≤60 years old or >60 years old), BMI (≤24 or >24), prior colonoscopy (yes or no), education level (university graduation, high school graduation, middle or elementary school), scheduled coloscopy time (AM or PM), indication (CRC screening, surveillance after previous colonoscopy or diagnostic), marital status(single or married), additional purgative(yes or no), compliance with dietary restrictions (yes or no) and compliance with purgative instructions (yes or no).

9.3 Sample size calculation

The rate of adequate bowel preparation in our endoscopic center is about 80%. We assume the application will raise the rate of adequate bowel preparation to 90%. To detect the difference with a significance level (α) of 0·05 and a power of 80% with a two-tailed test, we calculated that at least 394 patients will be needed for the study. However, from previous data, about 20% of patients may cancel their colonoscopy or have a failed colonoscopy, so we estimated that a total of 500 patients would be sufficient to detect a significant difference in the primary outcome.

10. Device description

'Qing Chang', version 2.0, provided by Henan Xuanweitang Medical Information Technology Co., China.

References

1. Bray F, Ferlay J, Soerjomataram I, *et al.* Global cancer statistics 2018: GLOBOCAN estimates of incidence and mortality worldwide for 36 cancers in 185 countries. *CA: a cancer journal for clinicians* 2018; **68**: 394-424.
2. Bretthauer M, Kaminski MF, Løberg M, *et al.* Population-Based Colonoscopy Screening for Colorectal Cancer: A Randomized Clinical Trial. *JAMA Intern Med* 2016; **176**: 894-902.
3. Sung JJ, Ng SC, Chan FK, *et al.* An updated Asia Pacific Consensus Recommendations on colorectal cancer screening. *Gut* 2015; **64**: 121-132.
4. Gimeno-García AZ, Hernandez G, Aldea A, *et al.* Comparison of Two Intensive Bowel Cleansing Regimens in Patients With Previous Poor Bowel Preparation: A Randomized Controlled Study. *The American journal of gastroenterology* 2017; **112**: 951-958.
5. Hassan C, Bretthauer M, Kaminski MF, *et al.* Bowel preparation for colonoscopy: European Society of Gastrointestinal Endoscopy (ESGE) guideline. *Endoscopy* 2013; **45**: 142-150.
6. Harewood GC, Sharma VK, de Garmo P. Impact of colonoscopy preparation quality on detection of suspected colonic neoplasia. *Gastrointestinal endoscopy* 2003; **58**: 76-79.
7. Liu Z, Zhang MM, Li YY, *et al.* Enhanced education for bowel preparation before colonoscopy: A state-of-the-art review. *Journal of digestive diseases* 2017; **18**: 84-91.
8. Hillyer GC, Basch CH, Basch CE, *et al.* Gastroenterologists' perceived barriers to optimal pre-

colonoscopy bowel preparation: results of a national survey. *J Cancer Educ* 2012; **27**: 526-532.

9. Spiegel BM, Talley J, Shekelle P*, et al.* Development and validation of a novel patient educational booklet to enhance colonoscopy preparation. *The American journal of gastroenterology* 2011; **106**: 875-883.
10. Tae JW, Lee JC, Hong SJ*, et al.* Impact of patient education with cartoon visual aids on the quality of bowel preparation for colonoscopy. *Gastrointestinal endoscopy* 2012; **76**: 804-811.
11. Abuksis G, Mor M, Segal N*, et al.* A patient education program is cost-effective for preventing failure of endoscopic procedures in a gastroenterology department. *The American journal of gastroenterology* 2001; **96**: 1786-1790.
12. Walter B, Klare P, Strehle K*, et al.* Improving the quality and acceptance of colonoscopy preparation by reinforced patient education with short message service: results from a randomized, multicenter study (PERICLES-II). *Gastrointestinal endoscopy* 2019; **89**: 506-513.e504.
13. Liu X, Luo H, Zhang L*, et al.* Telephone-based re-education on the day before colonoscopy improves the quality of bowel preparation and the polyp detection rate: a prospective, colonoscopist-blinded, randomised, controlled study. *Gut* 2014; **63**: 125-130.
14. Kang X, Zhao L, Leung F*, et al.* Delivery of Instructions via Mobile Social Media App Increases Quality of Bowel Preparation. *Clinical gastroenterology and hepatology : the official clinical practice journal of the American Gastroenterological Association* 2016; **14**: 429-435.e423.
15. Guo B, Zuo X, Li Z*, et al.* Improving the quality of bowel preparation through an app for inpatients undergoing colonoscopy: A randomized controlled trial. *Journal of advanced nursing* 2020; **76**: 1037-1045.
16. Wang SL, Wang Q, Yao J*, et al.* Effect of WeChat and short message service on bowel preparation: an endoscopist-blinded, randomized controlled trial. *European journal of gastroenterology & hepatology* 2019; **31**: 170-177.
17. Sinonquel P, Eelbode T, Bossuyt P*, et al.* Artificial intelligence and its impact on quality improvement in upper and lower gastrointestinal endoscopy. *Digestive endoscopy : official journal of the Japan Gastroenterological Endoscopy Society* 2021; **33**: 242-253.
18. Suzuki H, Yoshitaka T, Yoshio T*, et al.* Artificial intelligence for cancer detection of the upper gastrointestinal tract. *Digestive endoscopy : official journal of the Japan Gastroenterological Endoscopy Society* 2021; **33**: 254-262.
19. Hassan C, Mori Y, Antonelli G. AI everywhere in endoscopy, not only for detection and characterization. *Endoscopy international open* 2021; **9**: E627-e628.
