# Supplementary material for: Improving bowel preparation for colonoscopy with a smartphone application driven by artificial intelligence
Source: NPJ Digit Med. 2023 Mar 14;6:41. doi: 10.1038/s41746-023-00786-y (PMC10011797; doi:10.1038/s41746-023-00786-y)
Supplement: Supplementary file 2 — Reporting Summary [file 41746_2023_786_MOESM2_ESM.pdf]

nature portfolio

Corresponding author(s): Ping-Hong Zhou

Last updated by author(s): Jan 11, 2023

Reporting Summary

Nature Portfolio wishes to improve the reproducibility of the work that we publish. This form provides structure for consistency and transparency in reporting. For further information on Nature Portfolio policies, see our Editorial Policies and the Editorial Policy Checklist.

Statistics

For all statistical analyses, confirm that the following items are present in the figure legend, table legend, main text, or Methods section.

n/a	Confirmed	
☐	☒	The exact sample size (*n*) for each experimental group/condition, given as a discrete number and unit of measurement
☐	☒	A statement on whether measurements were taken from distinct samples or whether the same sample was measured repeatedly
☐	☒	The statistical test(s) used AND whether they are one- or two-sided<br>*Only common tests should be described solely by name; describe more complex techniques in the Methods section.*
☐	☒	A description of all covariates tested
☐	☒	A description of any assumptions or corrections, such as tests of normality and adjustment for multiple comparisons
☐	☒	A full description of the statistical parameters including central tendency (e.g. means) or other basic estimates (e.g. regression coefficient) AND variation (e.g. standard deviation) or associated estimates of uncertainty (e.g. confidence intervals)
☐	☒	For null hypothesis testing, the test statistic (e.g. *F*, *t*, *r*) with confidence intervals, effect sizes, degrees of freedom and *P* value noted<br>*Give P values as exact values whenever suitable.*
☒	☐	For Bayesian analysis, information on the choice of priors and Markov chain Monte Carlo settings
☐	☒	For hierarchical and complex designs, identification of the appropriate level for tests and full reporting of outcomes
☐	☒	Estimates of effect sizes (e.g. Cohen's *d*, Pearson's *r*), indicating how they were calculated

Our web collection on statistics for biologists contains articles on many of the points above.

Software and code

Policy information about availability of computer code

Data collection	Excel V16.
Data analysis	All statistical analyses were performed with SAS software (version 9.4).

For manuscripts utilizing custom algorithms or software that are central to the research but not yet described in published literature, software must be made available to editors and reviewers. We strongly encourage code deposition in a community repository (e.g. GitHub). See the Nature Portfolio guidelines for submitting code & software for further information.

Data

Policy information about availability of data

All manuscripts must include a data availability statement. This statement should provide the following information, where applicable:

- Accession codes, unique identifiers, or web links for publicly available datasets
- A description of any restrictions on data availability
- For clinical datasets or third party data, please ensure that the statement adheres to our policy

The data described in this manuscript may be made available upon reasonable request to Ping-Hong Zhou. (zhou.pinghong@zs-hospital.sh.cn).
The code may be made available upon reasonable request to Yan Zhu. (zhuyan1992521@163.com).

Human research participants

Policy information about studies involving human research participants and Sex and Gender in Research.

Reporting on sex and gender	Among all the 500 patients (47 in the control group and 253 in the AI–driven app group) in this clinical research, 260 (52%) patients are female.
Population characteristics	Overall, 578 patients were scheduled for colonoscopy examination during the study period. After excluding 54 patients who met the exclusion criteria or declined to participate, 524 eligible individuals were randomized to the control group or AI–driven app group. A total of 24 individuals canceled their colonoscopy appointment and did not reschedule. Ultimately, 500 participants, 247 in the control group and 253 in the AI–driven app group, were enrolled and included in the FAS analysis. After excluding patients who did not use the app correctly, 225 patients were included in the AI–driven app group for PP analysis.
Recruitment	Outpatients between 18 and 75 years of age scheduled for routine diagnostic colonoscopy were eligible for this study. For inclusion in the study, the patient was required to own a smartphone to access the app.<br>The exclusion criteria were as follows: (1) previous bowel surgery; (2) gastroparesis or gastric outlet obstruction; (3) known or suspected intestinal obstruction or perforation; (4) severe chronic renal failure (creatinine clearance <30 mL/min); (5) severe congestive heart failure (New York Heart Association class III or IV); (6) current pregnancy or breastfeeding; (7) toxic colitis or megacolon; (8) poorly controlled hypertension (systolic blood pressure >180 mm Hg and/or diastolic blood pressure >100 mm Hg); (9) moderate or massive active gastrointestinal bleeding (>100 mL/day); (10) major psychiatric illness; (11) allergy to the study purgatives; (12) inability to use a smartphone app; or (13) unable or unwilling to provide informed consent.
Ethics oversight	The entire study protocol was approved by the institutional review board of Zhongshan Hospital (B2020-297R).

Note that full information on the approval of the study protocol must also be provided in the manuscript.

Field-specific reporting

Please select the one below that is the best fit for your research. If you are not sure, read the appropriate sections before making your selection.

☒ Life sciences ☐ Behavioural & social sciences ☐ Ecological, evolutionary & environmental sciences

For a reference copy of the document with all sections, see nature.com/documents/nr-reporting-summary-flat.pdf

Life sciences study design

All studies must disclose on these points even when the disclosure is negative.

Sample size	The rate of adequate bowel preparation at our endoscopic centers is approximately 80%. We assumed that the app would increase this percentage to 90%. To detect this difference with a significance level (α) of 0.05 and a power of 80% using a two-tailed test, we calculated that approximately 394 patients were required for this study. Considering approximately 20% of patients cancel their colonoscopy appointment, so we estimated that a total of 500 patients would be required to detect a significant difference in the primary outcome.
Data exclusions	Overall, 578 patients were scheduled for colonoscopy examination during the study period. After excluding 54 patients who met the exclusion criteria or declined to participate, 524 eligible individuals were randomized to the control group or AI–driven app group. A total of 24 individuals canceled their colonoscopy appointment and did not reschedule. Ultimately, 500 participants, 247 in the control group and 253 in the AI–driven app group, were enrolled and included in the FAS analysis. After excluding patients who did not use the app correctly, 225 patients were included in the AI–driven app group for PP analysis.
Replication	Results here are reproducible because the patients are consecutively enrolled from multi-center.
Randomization	At the outpatient visit when scheduling the colonoscopy appointment, patients were interviewed by a research assistant not involved in the examination procedures. Written informed consent was obtained from all patients. The assistant explained the aims of the study and collected demographic and medical information on a data collection sheet. The eligible participants were randomized into the control group or the AI–driven app group (i.e., AI-driven bowel preparation group) in a 1:1 ratio by block randomization with stratification by center. The random allocation table was generated by SAS 9.4 software, and the randomization masking was implemented by opaque envelope. At least 50 patients were included in every center.
Blinding	Patients were informed of their group assignment and were required to not reveal their group assignment. All attending endoscopists were blinded to the patients' group assignment.

Reporting for specific materials, systems and methods

We require information from authors about some types of materials, experimental systems and methods used in many studies. Here, indicate whether each material, system or method listed is relevant to your study. If you are not sure if a list item applies to your research, read the appropriate section before selecting a response.

Materials & experimental systems

n/a	Involved in the study
☒	☐ Antibodies
☒	☐ Eukaryotic cell lines
☒	☐ Palaeontology and archaeology
☒	☐ Animals and other organisms
☐	☒ Clinical data
☒	☐ Dual use research of concern

Methods

n/a	Involved in the study
☒	☐ ChIP-seq
☒	☐ Flow cytometry
☒	☐ MRI-based neuroimaging

Clinical data

Policy information about clinical studies

All manuscripts should comply with the ICMJE guidelines for publication of clinical research and a completed CONSORT checklist must be included with all submissions.

Clinical trial registration	Chinese Clinical Trial Registry (ChiCTR2000040306).
Study protocol	The study protocol has been uploaded.
Data collection	Two endoscopists blinded to the entire clinical research reviewed the videos and assigned the BBPS score. If they did not reach a consensus, another senior endoscopist made the final decision.
Outcomes	The primary outcome was the percentage of patients with adequate bowel preparation. Adequate bowel preparation was defined as a total BBPS score ≥6 plus all segment scores ≥2. Two endoscopists blinded to the entire clinical research reviewed the videos and assigned the BBPS score. If they did not reach a consensus, another senior endoscopist made the final decision.<br>The secondary outcomes included the total BBPS score, BBPS score in each colon segment, rate of patients with perfect bowel preparation (BBPS score ≥8), compliance with dietary restrictions, compliance with purgative instructions, cecal intubation time, colonoscope withdrawal time, polyp detection rate (PDR), adenoma detection rate (ADR), and advanced adenoma detection rate (aADR). Compliance with dietary restrictions was defined as following the diet instructions and not consuming banned foods. Compliance with purgative instructions was defined as taking purgatives in the correct volume and at the correct starting time. PDR was defined as the percentage of patients with ≥1 polyp. ADR was defined as the percentage of patients with ≥1 adenoma. Advanced adenomas were defined as adenomas with an endoscopic size ≥10 mm, high-grade dysplasia, or villous features.<br>Patients were also asked about their sleeping quality during the bowel preparation process, rated as same as usual or worse than usual, and about whether they were willing to undergo bowel preparation for repeat colonoscopy in the future.
